# Non-targeted effects of photon and particle irradiation and the interaction with the immune system

**DOI:** 10.3389/fonc.2012.00080

**Published:** 2012-07-24

**Authors:** Thomas E. Schmid, Gabriele Multhoff

**Affiliations:** ^1^Department of Radiation Oncology, Klinikum rechts der Isar, Technische Universität München, Munich, Germany; ^2^Clinical Cooperation Group “Innate Immunity in Tumor Biology”, Helmholtz Zentrum München, Munich, Germany

**Keywords:** immune system, LET, bystander effect, abscopal effect, genomic instability

## Abstract

Ionizing irradiation is an important clinical approach to treat solid tumors. Modern radiation technologies aim to selectively kill tumor cells and protect the surrounding normal tissue. The standard paradigm for radiation effects in cellular systems involves damage of the DNA including DNA double-strand breaks, which are considered as most effective in destroying tumor cells. Due to their enhanced physical and radiobiological properties, high-linear energy transfer radiation qualities are of special interest in tumor therapy. Future radiation therapy strategies aim to utilize carbon ions to effectively treat highly aggressive tumors. More recently, evidence is emerging for non-DNA targeted effects of radiation, including mutations, chromosomal aberrations, and changes in gene expression, which can occur in cells that were not directly exposed to radiation. Radiation oncologists are only gradually beginning to appreciate the clinical relevance of radiation-induced bystander effects, genomic instability, and abscopal effects. Since these effects are sensed by the immune system, a combination of immunotherapy and irradiation presents a new therapeutic opportunity in the future.

## INTRODUCTION

The long-standing conventional paradigm for radiobiology for radiation effects in cellular systems has involved DNA double-strand breaks (DSBs) as the triggering lesions leading to mutation, cell death, and transformation. Depending on the linear energy transfer (LET) and dose, ionizing radiation causes a variety of different DNA lesions, including single- and double-strand breaks, DNA–protein cross-links, and DNA base damages (Bouquet et al., 2006). Ionizing radiation causes DNA damage either by a direct attack or indirectly via the production of free radicals and reactive oxygen species (Rothkamm and Lobrich, 2003). DNA DSBs are most fatal for cells because they can induce a complete loss or rearrangement of genetic material which results in cell death (Lobrich et al., 2005).

In recent years, high LET irradiation is gaining greater interest in tumor therapy, due to their improved physical and radiobiological properties. It is well-known that a spatial focused deposition of high energy by heavy ions can cause complex damage types (Jeggo et al., 2011). The oxygenation status has been identified as a pivotal factor for achieving locoregional tumor control by radiotherapy (Vaupel and Harrison, 2004; Vaupel et al., 2011).

The oxygen enhancement ratio (OER) decreases with increasing LET (Ferguson et al., 2000). This suggests a potential clinical advantage of high-LET radiotherapy with heavy ions compared to low-LET photon irradiation. Also mutations in the tumor suppressor gene p53, which are frequently found in different tumor entities, exert negative effects on the clinical outcome of radiation therapy. Irrespectively of the p53 and oxygenation status of carbon ions have shown efficacy in gliomas, human tongue, and lung cancer cell lines (Jakob et al., 2011). Therefore, future radiation therapy strategies aim to utilize carbon ions to treat highly aggressive tumors.

Recently, non-targeted irradiation effects that are not a direct consequence of the initial lesions produced by damages of the cellular DNA have been reported (Shiraishi et al., 2008). Since these effects are dependent on a functional immune system, it is important to protect the immune system against irradiation-induced damage. At present no clinically applied therapeutic options exist to protect the patient’s immune system. Most chemotherapeutic agents that are used in combination with radiotherapy to treat cancer exert immunosuppressive activities that might also suppress radiation-induced immunostimulatory effects (Shiraishi et al., 2008). Up to date, the effects of high-LET radiation on immune function have not been studied in detail. It is noteworthy that, unlike photon irradiation, particle irradiation may suppress the metastatic potential of cancer, suggesting that it may modify anti-tumor immunity via this treatment modality. Since high-LET cancer treatment using charged particles is performed only at very few sites worldwide, only little experimental information’s are available yet (Cai et al., 2009).

## RADIATION INDUCED NON-DNA TARGETED EFFECTS

**FIGURE 1:**
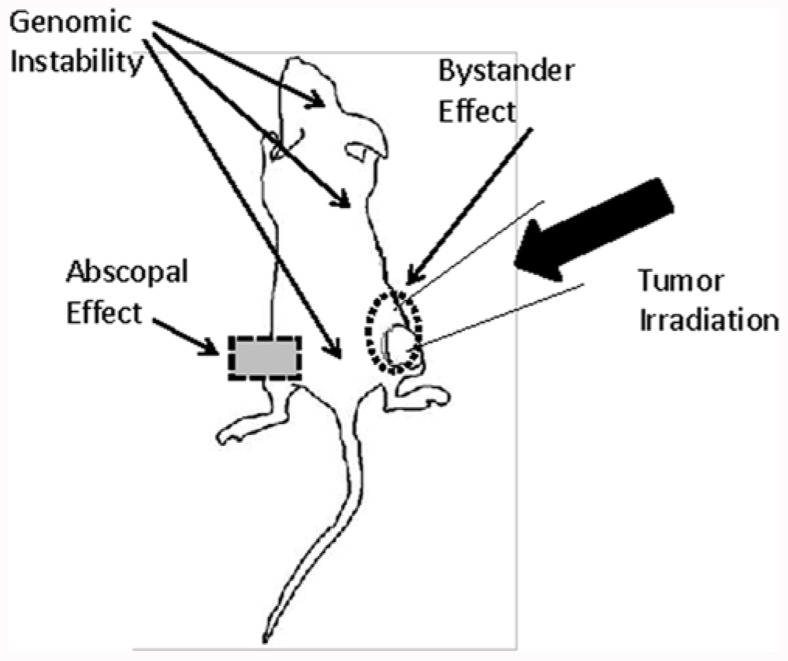
**The graph shows the different potential routes by which bystander, abscopal effects, and genomic instability may affect the outcome of radiation therapy in a tumor mouse model**. Radiation-induced DNA damage in the tumor can be amplified by bystander signals in cells residing in close proximity to the irradiation field. In contrast, abscopal effects and genomic instability exert distant and systemic effects.

Bystander effects, abscopal effects, and genomic instability are three phenomena which will lead to a paradigm shift in radiation biology (**Figure 1**). While the mechanisms underlying these effects are still not completely understood, it is very apparent that their implications are much wider than the field of classical radiobiology. The major adverse consequences caused by irradiation, such as initiation of secondary malignancies, are attributed to an inadequate repair in DNA damage in normal and tumor tissues. However, new studies have shown damage in cells that were not exposed to irradiation. These findings are explained by a potential interplay of irradiated and non-irradiated cells.

## BYSTANDER EFFECT

Since the discovery of X-rays in 1895, it was assumed that the deleterious effects of ionizing radiation such as mutations and carcinogenesis are mainly due to a direct damage of the DNA. Radiation-induced bystander effects are defined as biological effects in cells that are in close proximity to cells that have been irradiated (Hei et al., 2011). In 1992, Nagasawa and Little reported about an experimental system in which after exposure of 1% of the cells to densely ionizing particles, sister chromatid exchanges were observed in approximately 30% of the cell population (Nagasawa et al., 2003). The damage that occurred in non-irradiated cells has been described as the “bystander effect.” Unique microbeam facilities with the capacity to target subcellular areas within a cell such as the nucleus or the cytosol with a defined number of protons, photons or α-particles with high precision, play a pivotal role in a better understanding of the molecular mechanism of bystander effects (Hei et al., 2011). Using a microbeam in Columbia University, Wu et al. (1999) reported that a selective irradiation of the cytoplasm with four alpha particles results in killing of 10% of the cells and in increased gene mutations in the nucleus. It is speculated that either components of the cytoplasm or extracellular located components might be responsible for the observed increase in gene mutations in the nucleus.

Previous studies implicate that pro-inflammatory cytokine signaling is associated with *in vivo* chromosomal instability (Lorimore et al., 2008) and the involvement of COX-2 in the bystander response *in vitro* (Hei et al., 2008). The study of Lorimore et al. (2011) showed a connection of the bystander effect and the chromosomal instability that are mediated by signals involving COX-2 the initial enzymatic step in the metabolism of arachidonic acid to prostaglandins (Lorimore et al., 2011). Since NFκB is an important transcription factor for many signaling pathways including COX-2, it is likely that NFκB also participates in the bystander effect. There is clear evidence that alpha particle irradiation up-regulates the binding activity of NFκB via direct and bystander mediated effects (Zhou et al., 2008). Immune cells accumulate within and around tumors and cooperate with each other by utilizing specific cytokines. These results provide evidence that the COX-2 signaling pathway, which is essential in mediating a cellular inflammatory response, may be a critical signaling event for producing a bystander effect.

Importantly, *in vivo* experiments have demonstrated that cells of the innate immune system can be activated by ionizing radiation to produce pro-inflammatory mediators of genomic instability (Lorimore et al., 2008). Mutou-Yoshihara et al. (2012) showed that suppression of cytokine production was induced in the surrounding non-irradiated cells via the bystander effect (Mutou-Yoshihara et al., 2012). Bystander responses have been measured after exposures as low as a single proton or helium ion delivered to an individual cell. An important aspect is that the non-DNA targeted responses saturate with increasing dose to a single target cell (Prise et al., 2003).

The following conclusions can be drawn from experiments analyzing bystander effects: irradiation of the cytoplasm can induce genetic effects in the nucleus that was not directly exposed to radiation. It appears that the traversal of high-LET particles through the cytosol is more efficient than through the nucleus (Morgan and Sowa, 2009). Presumably, NF-κB, COX-2, and reactive oxygen species are involved in cytoplasmic irradiation-induced bystander effects.

## ABSCOPAL EFFECTS

The term “abscopal” is derived from the Latin prefix “ab,” meaning “away from,” and the Greek word “scopos,” meaning “target.” An abscopal effect has been defined as a reaction of cells within an organism that had not been directly exposed to irradiation, but cause tumor regression of the non-irradiated tumors (Postow et al., 2012). These responses indicate that the target size of the responding tissue is much larger than the irradiated field.

It is assumed that the abscopal effect is mainly mediated by an activation of the immune system via cytokines. The abscopal effect refers to distant effects observed after local radiation therapy (Shiraishi et al., 2008). Therefore, some investigators argue that abscopal effects should be termed as “distant bystander effects.” Although the immune system appears to be involved, the exact mechanisms of action of abscopal effects remain to be elucidated (Shiraishi et al., 2008).

Immune-mediated abscopal effects have been observed in mice with 67NR tumors after radiotherapy by studying the maturation status of dendritic cells (Formenti and Demaria, 2009). Radiation therapy seems to augment the ability of dendritic cells to capture and present tumor antigens and thereby mediating an anti-tumor-specific cytotoxic T cell response. Partial lung radiation experiments in rats demonstrated increased expression of tumor necrosis factor alpha (TNF-α), interleukin-1 alpha (IL-1α), interleukin-1 beta (IL-1β), interleukin-6 (IL-6), and transforming growth factor beta (TGF-β) in the shielded part of the lung that is adjacent but outside of the irradiation field (Langan et al., 2006). The generation of a sustained anti-tumor immune response at the irradiated tumor site, will not only determine the overall response of the irradiated tumor but also mediates an “abscopal effect” on the tumor sites outside of the treatment field (Formenti and Demaria, 2009). Apart from the activation of the immune system abscopal effects induce apoptotic signaling pathways. The irradiation of one tumor site resulted in the release of circulating tumor antigens and/or inflammatory factors that may then mediate an augmented immune response against non-irradiated, malignant lesions that express the same tumor antigens. It has been shown that local radiotherapy increases the activity of natural killer cells (Morgan and Sowa, 2007) that, as a result, can induce regression of non-irradiated tumors (Shiraishi et al., 2008). Importantly, irradiation of normal tissues of the mice did not induce abscopal anti-tumor immune responses. These findings suggest that radiation-induced stress and cell death responses of tumor but not normal cells are a prerequisite for the induction of specific anti-tumor immunity.

Clinical reports of abscopal effects after radiotherapy have been shown in different tumor types, including lymphoma, melanoma, and a variety of carcinoma (Hiniker et al., 2012; Postow et al., 2012). Abscopal effects are not restricted to ionizing irradiation but also have also been observed after surgery, hyperthermia, and laser therapy (Martin et al., 2011). It is assumed that abscopal effects require secreted factors to mediate systemic immune effects (Morgan and Sowa, 2009).

A better understanding of abscopal effects might improve the clinical outcome of radiotherapy.

## GENOMIC INSTABILITY

Although ionizing radiation is known to induce secondary malignancies in many tissues, the underlying mechanisms at the cellular and molecular level are not completely understood. An attractive hypothesis is that radiation induces “genomic instability” in a subpopulation of cells harboring multiple mutational events that are required for the transformation of a normal tissue into an invasive tumor (Huang et al., 2003). DNA damage, as the biological consequence of irradiation, is observed within minutes post exposure. However, indirect effects of irradiation including genomic instability and carcinogenesis occur after months and years following irradiation (Morgan, 2003). Genomic instability is defined as an increased rate of acquisition of alterations in the genome, manifesting as chromosomal aberrations, micronucleus formation, gene mutations, and aneuploidy (Morgan, 2003). The current hypothesis to explain radiation-induced genomic instability is that radiation initiates sublethal damage in one cell that is communicated to other cells and as a result causes a destabilization in the genome (Huang et al., 2003).

Lorimore et al. (1998) demonstrated genetic instability in the surviving fraction of shielded, non-irradiated tumor cells residing in close proximity to cells exposed to alpha particles. These data clearly demonstrated that genomic instability could be induced by an interaction of irradiated and non-irradiated cells. Radiation-induced chromosomal instability appears to involve a significant epigenetic component and a link between non-targeted bystander effects resulting in chromosomal instability in non-irradiated cells (Huang et al., 2003; Lorimore et al., 2008). Intercellular signaling, production of cytokines, and free radicals are features of inflammatory responses that have the potential for both bystander-mediated effects and genomic instability (Lorimore et al., 2008).

The severity of genomic instability is influenced by the LET (Limoli et al., 2000, Smith et al., 2003). While there is a clear dose response for direct radiation effects immediately following exposure, there is no typical dose response for the delayed indirect effects of exposure to irradiation (Limoli et al., 2000). However, it is known that after high-LET radiation chromatid aberrations are more prevalent than chromosome aberrations.

As a clinical implication, genomic instability can serve as a marker for an increased risk to develop secondary malignancies after radiation therapy. Of particular interest is the observation that transmissible instability can be induced in somatic cells from normal individuals by exposure to ionizing radiation, leading to a persistent enhancement in the rate at which chromosomal aberrations arise in non-irradiated cells after many generations of replication (Little et al., 2002).

## CONCLUSION

Current anti-cancer modalities such as surgery, chemo-, and radiation therapies have only limited success in the cure of solid tumors in advanced stages. During the past decade progress has been made in the understanding of the fundamental mechanisms and biological significance of the immune system in the control of cancer. The major challenge in the field is to understand the various molecular mechanisms involved in non-DNA-targeted irradiation effects that counteract tumor-related signaling pathways. Irradiation-induced abscopal and bystander effects have been shown to stimulate the immune system of cancer patients and thus might exert beneficial effects. A better understanding of the immune-modulatory effects of heavy-ion beam treatment will help to develop innovative and more effective strategies for charged-particle therapy in clinical settings.

## Conflict of Interest Statement

The authors declare that the research was conducted in the absence of any commercial or financial relationships that could be construed as a potential conflict of interest.
